# Iranian staff nurses' views of their productivity and human resource factors improving and impeding it: a qualitative study

**DOI:** 10.1186/1478-4491-3-9

**Published:** 2005-10-08

**Authors:** Nahid Dehghan Nayeri, Ali Akbar Nazari, Mahvash Salsali, Fazlollah Ahmadi

**Affiliations:** 1 Faculty of Nursing, Tehran University of Medical Sciences, Tehran, Iran; 2 Faculty of Medicine, Tarbyat Modarres University, Tehran, Iran

## Abstract

**Background:**

Nurses, as the largest human resource element of health care systems, have a major role in providing ongoing, high-quality care to patients. Productivity is a significant indicator of professional development within any professional group, including nurses. The human resource element has been identified as the most important factor affecting productivity. This research aimed to explore nurses' perceptions and experiences of productivity and human resource factors improving or impeding it.

**Method:**

A qualitative approach was used to obtain rich data; open, semi-structured interviews were also conducted. The sampling was based on the maximum variant approach; data analysis was carried out by content analysis, with the constant comparative method.

**Results:**

Participants indicated that human resources issues are the most important factor in promoting or impeding their productivity. They suggested that the factors influencing effectiveness of human resource elements include: systematic evaluation of staff numbers; a sound selection process based on verifiable criteria; provision of an adequate staffing level throughout the year; full involvement of the ward sister in the process of admitting patients; and sound communication within the care team. Paying attention to these factors creates a suitable background for improved productivity and decreases negative impacts of human resource shortages, whereas ignoring or interfering with them would result in lowering of nurses' productivity.

**Conclusion:**

Participants maintained that satisfactory human resources can improve nurses' productivity and the quality of care they provide; thereby fulfilling the core objective of the health care system.

## Background

In most health care organizations, nurses are the largest work group and play a major role in the organization's success. Hence nurses' productivity affects an organization's success by influencing organizational total factor productivity (TFP) [1]. Health care organizations cannot succeed without productive nursing staff [2]. But recent studies suggest that nurses no longer feel their work is valued and are concerned with their productivity [3].

Although nurses are concerned about declining levels of effective care and productivity [4], staff productivity rarely has been assessed within the health care organization of Iran and little is known about factors that affect nurses' productivity. The complexity associated with the impact of human resources on nurses' productivity is one of the factors least studied.

Managers' efforts to promote productivity have been mostly ineffective and have resulted in too many changes and personnel frustration without improving patient care [5]. Assessing nurses' viewpoint on productivity should be the first step towards improving nurses' productivity.

### Theoretical and empirical approaches to productivity

Productivity is defined as the ratio of outputs to inputs or as the relationship between inputs and outputs. McConnell suggested that comparing output to input was similar to comparing apples with oranges, because the two are often so different [6].

In light of traditional economic definitions of productivity as the numeric cost ratio of outputs to inputs, administrators in health care have focused on quantifying nurses' work in economic input-output terms [7]. Within the discipline of nursing, some viewed nurse productivity as a measure of the efficiency with which the input of nursing tasks and other labourers' tasks, materials and equipment were converted into goods and services delivered within the health care system [3,7].

Also, nursing productivity was defined as equilibrium between demand for and supply of services and managing cost structure of a system by integration of financial and clinical processes and providing good care in a cost-effective manner. Some researchers have defined productivity as managing staffing (depending on census and patient acuity) to meet budgeted standards [6]. Others view nurse productivity as the ratio of output produced compared with resources consumed and proposed that nurse productivity can be measured at the hospital unit, departmental or divisional levels [7,8].

Employee productivity was also measured by hours of care per day and salary dollars per procedure. Sometimes acuity also was factored in. Productivity was also measured by budgetary standards set by organizations, or through community or national norms. Recently, in organizational redesign strategies, productivity measures have been set by measuring the time necessary to do each job, and incorporating acuity standards [5].

Another measure of nurse productivity that has become prevalent and is used by many hospitals is the popular "hours per patient day" (HPPD) unit of measure for nurse productivity [7,9]. Nursing care hours have been further delineated into direct and indirect care hours, and sometimes have focused on costs [6].

Despite that term nursing productivity is an old concept used daily by nurse executives, there is little clarity and consistency in how it is used [6]. Also, these different definitions of productivity lead to measurements that cannot be compared [5]. In a nursing context, productivity has been used exclusively in terms of resources used – based on economic theories of productivity- while certain significant outcomes of nursing practice have not been considered [10,11]. In addition, the emphasis of economic theories of productivity is on measuring administrative systems rather than on what has been accomplished [11]. In business literature and research, productivity has usually been discussed in terms of hypothetical variables that could improve the outcome.

For instance, researchers (1993) reported that employees with higher levels of job satisfaction and skills directly related to their jobs had significantly higher productivity ratings than their co-workers [12]. Another study revealed that practices such as performance appraisal had a strong effect on productivity [13]. In addition, training programmes for new employees increased their productivity [14]. A number of researchers (McCloskey and McCain 1988, McNeese Smith 1995, 1996) have assessed factors affecting nurses' productivity [15-17], while Grosskopf, Margaritis and Valdmanis (2001) evaluated effects of teaching on hospital productivity [18]. Hall (2003) assessed the contribution of knowledge and skill, and factors such as organizational trust and commitment on nursing productivity [3]. Curtin (1995) described how patient classification could be used to improve staff productivity [11]. However a concept analysis done by Holcomb, Hoffart and Fox (2002) revealed the complexity of the concept and its measurement [6]. Even though it is essential to understand care providers' views about productivity, only one study (McNeese Smith 2001) examined in detail how nurses viewed their own productivity.

However, no studies have examined nurses' productivity in Iran; findings of studies undertaken in other countries may not be directly applicable to the Iranian context due to significant cultural and socioeconomic differences.

Identifying factors that affect productivity from the nurses' viewpoint leads to an understanding of how best to improve productivity. Therefore, this study aimed to highlight nurses' perception of their own productivity and factors affecting it. It attempted to address questions such as "What are nurses' perceptions of their own productivity?" and "Which factors improve or diminish nurses' productivity?"

To address the former questions a qualitative methodology was adopted. It was considered that this was the most suitable approach to address the complexities of the subject of this study, which a quantitative approach may fail to fully elucidate. A qualitative approach was used as an important and essential first step in understanding the participants' insiders' view and their perceptions. This method resulted in a set of themes and categories about the perceived productivity and human resource elements aiding or impeding nurses' productivity.

## Method

Semi-structured interviews were used to gather data covering nurses' perception of their own productivity and factors influencing it. This approach provides understanding of any phenomena at a deeper and richer level that could not be reached through a quantitative approach [19]. Content analysis has been applied to transcriptions and interviews for the purpose of understanding human behaviours within various contexts, as it is an unobtrusive means of analysing interactions and provides insight into complex models of human thought [21].

In the current study, the content analysis method was used to identify categories and subcategories in participants' descriptions. Qualitative content analysis elicits contextual meaning in context through the development of emerging themes. This method consisted of identifying, coding and summarizing the concepts and themes, consistent with established qualitative data analysis methods [20]. Also this is a method that uses a set of categorization procedures for making valid and replicable inferences from data (text or images) to their context. Researchers analysed the presence, meanings and relationships between words and concepts, and subsequently made inferences about the messages within the texts.

This method was particularly suitable for productivity, as the outcome of social interactions lends itself to content analysis of the core aspects of social interaction. Furthermore, this method allowed a closeness to text that can alternate between specific categories and relationships. This method enabled us to explore rich data and allowed an interpretive understanding of what was going on.

### Sampling and data gathering

Sampling was targeted based on a set of predetermined criteria. For instance, in the current study, the researchers made preliminary sampling decisions to recruit staff with a minimum of five years' nursing experience working in hospitals affiliated to Tehran University. It was considered that the participants would have sufficient work experience to enable them to analyse factors affecting their productivity and its process. The sampling was based on the maximum variant approach.

Sampling started with a nurse with 29 years' experience and then extended to other nurses, managers or supervisors in the same teaching hospital or others. The setting was education hospitals affiliated to Tehran Medical University.

In this study data collection and analysis proceeded concurrently with the development of themes related to the reality of the nurses' productivity. Sampling continued until saturation was reached: this was when no new categories or subcategories emerged [22].

### Participants and interviews

Initially, the researcher contacted each of the potential participants and explained the objectives of the study. If they agreed to take part in the research, interview time and setting were arranged. Semi-structured interviews, based on clear guidelines, were usually carried out in a rest room on the ward. Participants were initially asked to describe a typical working day. They were subsequently asked about their perception of nurses' productivity and asked to highlight experiences where they had felt productive as a nurse and identify factors that had a positive or negative impact on their productivity. Each interview was approached individually, guided by participants' responses while covering the core research questions.

The selection criteria were that they be staff members with more than five years of nursing experience who worked full-time in hospitals covered by the Ministry of Health and Medical Education in Tehran, Iran. Any nurse who met this qualification was considered a potential participant.

A total of 26 participants in various positions were interviewed. Interview sessions lasted from 30 minutes to 70 minutes. Interviewing continued on all shifts, over a three-month period, until the information from the last four interviews with new participants was becoming repetitious and thus saturation of the data seemed to have been achieved.

### Ethical considerations

This study formed a part of a PhD thesis in Tehran Medical Science University. The ethics committee of the University reviewed and corroborated its ethical considerations. All the participants were informed of the purpose and design of the study, and that their participation was voluntary and would be treated with confidentiality. Participants were asked to sign a form confirming informed consent prior to taking part in the study. Audiotaped, semi-structured interviews were conducted in private rooms and solely by the researcher. In addition, permission was obtained from hospital directors and head nurses for the nurses to be interviewed in their work setting. They were assured that participants could cease participating in the study at any point.

### Data analysis

All interviews were audiotaped. Analysis of data was based on overall impression, reading and re-reading of transcripts. Analysis consisted of identifying, coding and summarizing the concepts and themes, consistent with established qualitative data analysis methods [20]. Data were analysed by the constant comparative analysis method and coding process. Tapes were transcribed and data were broken down into discrete parts and codes were noted next to phrases, words or comments in the text. Then codes were sorted into categories and redefined into further focused categories.

All data were grouped within the categories according to their "fit". When required, the researcher returned to the field to extend categories. For instance, some of the interviews and codes indicated that the education process has a significant impact on the group's productivity, and subsequent sampling focused on this factor. Also, some of the interviews and codes indicated that nurse managers – especially supervisors and matrons – have a significant impact on the group's productivity through adopting appropriate qualitative and quantitative strategies to address staffing issues. In addition, they have valuable experience about group productivity. They worked as nurses in the wards before becoming managers. Throughout the process the researcher investigated the causal conditions arising from the data.

### Validity and reliability

To maintain trustworthiness of the conclusions, Lincoln and Guba's four criteria were used. This in quantitative research is equivalent to empirical positivistic criteria (validity and reliability). The four trustworthiness criteria are credibility, confirmability, transferability and dependability [22].

Credibility was enhanced through prolonged engagement with participants, the achievement of saturation, sampling approach (maximum variant sampling). In particular, participants' revision of coding – member check – supported credibility. After data analysis participants were provided a complete transcript of their coded interviews with emergent themes. They verified whether the codes and themes matched their answers. Maximum variant sampling also validated the confirmability of data.

Also reliability of study results was further enhanced when other researchers in the field were asked to review the interviews and the coding process. They were three expert supervisors and two other faculty members experienced in qualitative research. They checked a large number of the transcripts of interviews and coded them. There was close agreement (90% or higher) between the resultant coding achieved by a number of researchers.

Furthermore, the results were discussed with other nurses who did not take part in the research but who confirmed the soundness, fitness and transferability of the results. This confirmed transferability of results.

## Results

Data saturation was reached with 26 participants. Twenty-four of the participants were female, 30 to 56 years of age. All of them had bachelor's or master's degrees. They had between 5 and 29 years of work experience with a mean of 17 years. Participants were clinical nurses, head nurses, supervisors, nurse managers and nursing educators.

The findings of this study indicate that human resources is the most important factor affecting nurses' productivity. Other factors included managers' role, organizational structure and culture, social factors, financial security and the nature of the nursing work undertaken.

In this paper, we have discussed two categories – human resources and productivity – that emerged from content analysis. The human resource category included several subcategories. Categories and subcategories of nurse productivity that emerged are reported, together with certain extracts from the interviews to highlight nurses' experiences and the manner in which they have has been communicated.

### Definitions of productivity

Participants defined their productivity from different perspectives. Although participants used different terms and occupied various positions, they were referring to the same concept and themes that were highlighted in almost all interviews. For instance, while some used the term "usefulness" others chose terms such as "effectiveness", "being efficient", "providing high quality care" and "being at the bedside and providing good care" to refer to productivity. We classify these words into two categories: the quantitative aspect, which is equivalent to efficiency, and a qualitative feature that is equivalent to effectiveness.

Most of the participants referred to productivity as a qualitative feature, while some of them implied both quantitative and qualitative aspects. In certain cases, participants referred to productivity based on prerequisites or outcomes relating to productivity. However, the majority of the participants in the current study considered productivity as equivalent to "helpfulness, effectiveness for patients and providing high quality care". Therefore, productivity in these nurses' opinion means to be effective for the patients, and that they feel they are highly productive when "they focus on taking care of patients". Table 1 shows productivity from the Iranian nurses' viewpoint.

**Table 1:** Productivity from the Iranian nurses' viewpoint

**Quantity**	**Quality**	**Prerequisites**	**Outcome**
To be efficient	To be effective	Commitment to the organization	Patient satisfaction
To use time properly	Providing high-quality care	To be conscientious	Personnel satisfaction
Accomplishing all the necessary tasks	To be useful to patients	To be responsible	Organization profit
Doing work correctly	Being at the patient's bedside (accessibility for patients)	To be careful	
Working with high ability	Doing the right work	To be aware	
Working with minimum facilities	Solving patients' problems		

One of the participants stated: "In my opinion, our productivity is increased when we can provide our clients skilled care as well as providing a condition in which the patients are treated and possibly regain their full health" (a primary-care nurse). A staff nurse said: "I feel productive when giving good care and meeting their needs ". Another nurse said she felt a sense of productivity "when my patients feel satisfied at the end of the shift".

Most of the participants believed that nurses do not have high productivity and cited a number of reasons for this; others stated that under present conditions nurses could not be expected to do a lot more. One of the participants said: "I think considering what we studied and what is expected of us, nurses' productivity is about 20 %"(a head nurse). She added that nurses' energy was spent mostly on administrative tasks, which has an adverse impact on their productivity and effectiveness for patients.

Thus from the participants' viewpoint, existing productivity was affected by the lack of resources, especially the shortage of experienced personnel, as well as having to perform non-nursing administrative tasks.

Nurses in this study also believed that lack of equipment, shortage of personnel and disproportionate nurse-to-patient ratio have negative effects on productivity. For example, one participant said: "Considering the existing facilities and very high numbers of patients, nurses could even be considered to have high productivity levels compared with other professionals, but if compared with developed countries, nurses' productivity in Iran is poor" (a nurse with 20 years of experience). Overall a range of factors – especially shortage of nurses, workload and inexperienced personnel – all have a negative impact on nurses' productivity.

Nurses believed that they use only 15–20% of their productivity as they have to perform certain duties that they felt they should not perform. That is to say, 80% of their productivity is wasted, as it is not directed to patient care.

One of the participants with extensive clinical, management and nursing education experience, referred to unfavourable conditions for nurses, saying: "Productivity promotion requires certain conditions of which even the minimum do not exist for Iranian nurses. Even though the number of nurses is probably 30%–50% of the minimum required, the expectations are very high." Finally participants believed that most of the factors that hinder their productivity can be altered by managers through modification of human resource elements.

### Human resources

The participants most frequently referred to human resource elements, and believed them to be the most important factor affecting productivity. Although the impacts of such a factors are widely known by participants – managers and nurses – alike, they believed that human resource impediments are still widely prevalent in all spheres of their work. The subcategories of human resources are: personnel needs and nurse staffing, staff expertise and experience, work coordination and teamwork, and effects of present staffing.

#### Personnel needs and nurses staffing

In the participants' view, one of the factors facilitating achievement of higher productivity is an appropriate staffing level. They believe this should be assessed in the context of nurses' functions and role within the system. It is possible to evaluate the appropriate staffing requirements only if nursing workload, patient needs and their required care and the level of non-nursing administrative tasks are fully considered. Participants stated that all the latter factors need to be considered, if the correct staffing levels are to be achieved and personnel shortage and excessive workloads are to be avoided. A supervisor said: "nursing workload is not assessed systematically; decision-makers only consider routine tasks. Our hospitals put additional demands on nursing staff from direct patient care to voluminous clinical and medical documentation... but these are not assessed when personnel needs are estimated for wards".

While one of the factors facilitating achievement of higher productivity is appropriate staffing level, participants believed that in most cases the wards are facing a shortage of professional and assistant nurses. As one surgical nurse said: "We are usually two nurses for 35 patients, without doubt we cannot supply a high-quality care and to be productive". Another nurse said: "We can only give the drugs and monitor vital signs ... a routine work, we cannot provide effective care because there is so much work". One of the general nurses said: "I don't feel productive when it is too busy and work overload, and I can't help all my patients". Another nurse said: "I know that we don't have the time to provide all the care correctly".

Participants believed this shortage is in turn due to a non-systematic approach, which fails to fully take into account factors such as physical structure of the ward, facilities, nurses' experience and their skills, nature of the care patients require and admission procedures, as well as circumstances of other hospital services such as the pharmacy and catering section. Participants stated that all of these factors need to be considered if the correct staffing levels are to be achieved and personnel shortage and excessive workloads are to be avoided.

For instance, one of the participants highlighted the effect of the physical structure of the unit, which has an impact on the number of required personnel. She stated "This unit has a very long corridor. If you are at one end of the unit and need something, you have to walk a long distance to reach the nursing station and this affects the number of required personnel" (chief nurse of a surgery ward).

Nurses cited numerous experiences highlighting shortcomings for patients and the nursing staff alike resulting from shortages of human resources. For instance, one said: "There have been situations in which we have had three ICU cases, three cases of bedsores and patients needing tube feeding and suctioning. Sometimes we have to spend one hour on a patient's dressing. In these circumstances, how can nurses provide adequate care as and when needed? When one has to complete work that requires four members of staff, this invariably gives rise to immense stress". Another participant said: "The pressure of work and overloading of responsibilities made nurses so busy that they seldom have enough time to be productive – that is, we cannot provide excellent care".

Personnel shortage has also resulted in patients' care being delegated to non-nursing personnel and the patients' family, who probably lack sufficient skills. Finally this will result in a decline in productivity and quality of care and cause patients' dissatisfaction. One participant said: "We give patients' care to their family because we cannot meet all the patients' needs...we seldom have enough time ... we are very busy".

In participants' views, lack of staff replacement cover in cases where personnel are on leave or when staff have completed their Free Education Compensation Period (FECP) as well as periods when no FECP persons are referred to hospitals, are other factors that increase staff work pressures. Furthermore, a reluctance of FECP personnel to work in centres with no NET is another parameter that exacerbates hospital staff shortages and increases the workload for the existing staff.

Delegating nurses to nursing roles not in line with their qualifications or nurses' working in other sections restricts appropriate human resource distribution, resulting in low nursing productivity and inappropriate patient care. One nurse manager said: "Our NETs work everywhere (pharmacy, etc.) except as a nurse on the hospital ward, but when assessing the staffing levels within the wards, they are included in the nursing statistics".

#### Selection procedures

As nursing aims to serve the public it needs active and motivated personnel. Correct selection of nurses and nursing students can promote nurses' productivity, according to respondents. In the participants' view, selection of capable nurses is possible only through setting proper selection procedures and adhering to them in order to ensure nurses are selected based on merit. One of the supervisors said: "Employment regulations should be closely adhered to, but here the people who perform the selection process are by no means capable."

#### Staff expertise and experience

Participants cited nursing personnel's skill and experience as one of the factors resulting in productivity improvement. Also, new-staff orientation and assigning newly qualified staff to work alongside experienced personnel are two important elements aiding nursing team productivity, in the participants' viewpoint. Nevertheless participants, especially managers, have pointed out the shortcoming in this respect and said they believe that staff lack of knowledge is a significant barrier to being productive and providing a high quality of care. They cited application of knowledge and skill in special wards as the sign of effective care. They believed they were more productive in such wards for this reason alone.

Participants, especially those in management positions, argued that employing recently graduated staff could diminish the overall system's quality because of inadequate experience. One participant said: "All shifts were covered by newly graduated personnel who are novices and inexperienced. It takes them so long to adjust and learn clinical work, and the patient suffers the consequences of the shortcomings" (a manager of a paediatrics hospital).

Participants believed that hiring newly graduated staff who have little experience and skill and a high ratio of these staff compared to experienced personnel, as well as frequent personnel turnover, are among factors that lead to low overall skill and experience level within the ward. They believed these factors contribute to increased pressure on the nurses and managers, and ultimately lead to complications for the patients. One participant commented: "It is not only the number of staff that has an impact on productivity, but the skill and experience of the personnel also play an important role"(a staff nurse).

Participants claimed that permanent employment was a significant positive factor contributing to increased skill, experience and a more responsible approach towards patients, and in the organization as a whole leads to improved productivity.

#### Work coordination and teamwork

In the participants' viewpoint, not only is a systematic assessment of human resource requirements lacking, but one of the factors impeding productivity and effective care is a lack of coordination among different workgroups. Un-systematic patient admission creates an imbalance in the nurse/patient ratio within the ward. Informants believed that this is caused by a lack of harmony between the physicians and hospital managers as well as uncoordinated patient transfer from other hospitals and emergency centres. One participant said: "As a result of uncoordinated patient transfer from other hospitals, our ICU beds are 110% occupied; while our staff are sufficient for 80%–90% occupancy" (a supervisor of infant care).

In addition to the number of personnel, informants believe that appropriate communication between team members is an important factor contributing to their productivity. Effective communication can improve group performance and reduce the effect of excessive workload caused by personnel shortage on tolerance levels and coordination within the ward, resulting in higher productivity. Participants frequently referred to their close relationships with managers, which they believed resulted in higher productivity. For instance, one participant said: "There should be strong relationships between the personnel in a trusting environment. This would enable staff to discuss and address any possible errors at an early stage to avoid further damage" (a chief nurse of a unit). Another nurse said: "We worked under difficult conditions within the emergency unit but sound working relations between personnel and in particular with the unit manager enabled us to work effectively." However, participants believed shortage of staff and a heavy workload can have a negative effect on interpersonal relations.

#### Effects of present staffing

Participants believed that shortage of nursing staff has a negative impact on both the productivity of the nurses and the effectiveness of care. Moreover, this leads to certain significant care procedures' being overlooked and increased errors and necessitates involving unsuitable staff or patients' friends or family members in the care process.

Personnel shortage hinders effective management processes such as comprehensive personnel performance appraisal, which is a prerequisite of improving productivity and patients' care. In this context, a head nurse commented: "If I criticize the quality of nursing care they respond by pointing out that there is merely one nurse for every 20 patients. They obviously have a point, and I have to overlook certain shortcomings".

Staff shortage impedes nurses' productivity directly and indirectly and creates a difficulty for both patients and nurses. Participants stated that staff not practising learnt principles, not providing sound nursing care, overlooking patients' education and effective communication with patients, not assessing the patients' condition and not solving patients' problems are all attributable to staff shortage. In addition, participants have stated that providing merely routine patient care devoid of attention to individual needs, increased risk of practical errors and sometimes even being unaware of the presence of certain patients' within the ward until their discharge are all due to personnel shortage and unsuitable human resources.

The informants believed that personnel shortage not only adversely effects patient care, but also over a period of time excessive work pressure and undertaking a range of duties results in nurses' loss of knowledge and motivation, exhaustion, burnout, severe stress and eventually leads to staff leaving the profession. One of the participants said "It gives us a sense of burnout... masses of things you constantly feel you should be doing, looking after the patient, you have to do the documentation. It is a high pressure. I don't sense I am productive; I think I am burned-out, tired".

However all the participants believed that appropriate staffing level is the most important factor facilitating their productivity. When asked how to improve productivity, all the participants indicated that provision of human resources to a high standard in all nursing ranks, as well as proper personnel planning and organization, would enhance productivity. One said: "One way to ensure that care is accomplished properly and completely is to have adequate staff" (first staff of a ward). Also, participants believed that most of the factors that hinder their productivity can be altered by managers through modification of human resource elements.

Ultimately participants believed that achieving increased productivity requires certain conditions, but that the present circumstances lack even minimum criteria and than an inappropriate human resources pattern is a major obstacle in this respect. One said: "When talking about productivity of a group, certain basic conditions should be in place before they could be expected to perform to the required standard. Currently only one nurse is assigned to care for five patients in ICU whereas they allocate three nurses for two or three beds in other countries" (a participant with clinical and management experience).

## Discussion

In this study, nurses described productivity as "being effective for the patients" and said they feel they have high productivity when "focused on taking care of patients".

**Figure 1:**
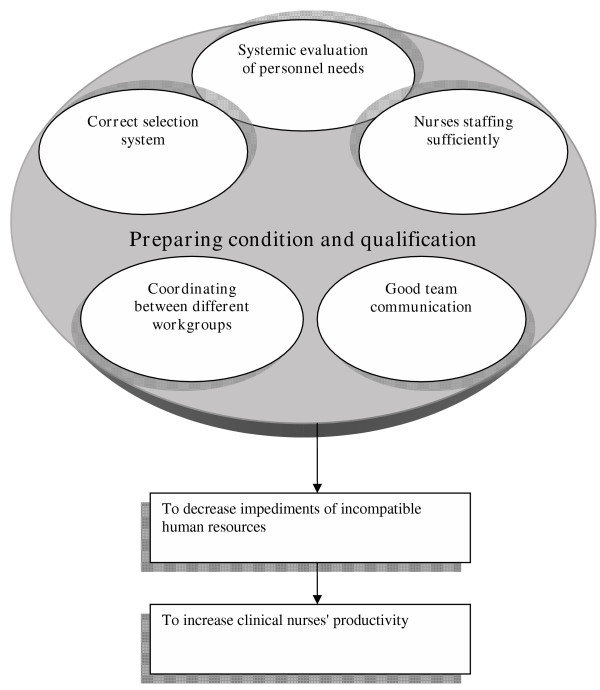
Nurses' productivity and human resources.

The finding of this research has been presented in a diagram (Fig. 1). The concepts within this diagram include systematic evaluation of staff numbers required; appropriate, adequate and regulated selection process; supplying staff of different ranks; patient admission coordinated with the head nurse; and appropriate working relations within teams. Adhering to these themes on the whole creates the potential for increased productivity, which in turn reduces negative impact of inappropriate human resource provision.

The findings of this study indicated that productivity from nurses' perspective differs from the adopted models suggested by industry and economy, which focus on output rather than outcome. The output is amount produced from input. This is a quantitative fragment but outcome is all the results of a work process.

The key point here is that although productivity from an administrative organizational perspective is represented by cost-effectiveness, nurses often assess their own productivity by quality of care provided to patients. This fundamental difference between nurses' perception and that of managers results in different assessments. In this study, nurses believe that to improve productivity there should be adequate and qualified human resources, while organizations consider increased productivity in terms of limiting permanent employment, imposing compulsory overtime with the least financial benefit to workers, hiring FECP staff and decreased cost.

Even though productivity has so far been considered by organizations and managers from a quantitative perspective (efficiency), nurses often emphasized the qualitative dimension and their own effectiveness. Participants consider productivity in the context of the whole care process and its outcome. This gap reflects the different perspectives of those who decide nursing budgets and often are not nurses themselves and that of nurses, who are in direct contact with the patients. Although for an organization's managers the logic of cost-efficiency prevails, for nurses providing high-quality care is the major goal that not only results in patients' self-sufficiency and their return to full health but promotes the profile of the nursing profession as a whole.

Accordance to results of this research, management experts believe that methodology of staffing is a systematic process that is used to evaluate the exact numbers and type of personnel needed to provide the standard care in an organization [23]. Researchers believed that although many staffing studies use various methods to quantify the volume of tasks that nurses must provide for patients, few consider the impact of non-staffing elements on nurses. They did not consider nursing work context as a system. Nurses do not care for patients in isolation but within complex organizations that make great demands on them [4]. Analysis of nurses' work structure based on 100 000 individual observations in two hospitals over a seven-year period revealed that about two-thirds of nurses' time was spent on indirect care and merely a third was dedicated to direct care [8].

Also, researchers believed that nurses' workload is dictated by factors such as nursing standards, nurses' experience and skill, organizational strategies and procedures, available equipment and the activities of other members within the health care team [24]. These are according to results of this research.

Former studies have shown that patterns and personnel combinations as well as skill combinations and education levels of the personnel positively affect productivity [25] and the fact that a higher ratio of experienced nurses decreases hospital operational costs [26]. Researchers believe that chief executives and their management team can influence productivity by hiring skilled, industrious and capable people [27]. Hall (2003) concluded that the future of nursing depends on the knowledge and skill of the nursing workforce and the availability of adequate numbers of nurses for the health care sector [3]. Such sentiments agree with findings of this research that staff expertise and work experience positively affect productivity.

Hall (2003) has stated that since individuals differ in intelligence, skills, motivation and personality it is essential that organizations determine what qualities they are seeking when recruiting new staff, in order to ensure that they can function effectively within the organization [3]. According to the results of research, Huber (2000) also believed that planning and staffing levels affect nurses' career and work conditions, workload, personal life and morale. Also she confirmed that security and quality of care to the clients are affected by staffing [28].

Work coordination and teamwork is one of the themes that emerged from the data. Houser (2003) demonstrated that effective teamwork and greater expertise can have a major influence on the outcome of patient care [4]. Curtin's research findings showed the relationship between teamwork and prevention of complications [11]. Also, previous research suggested that the quality of communication among disciplines is important for achievement of positive outcomes [29].

Another finding of this study was that nurses cannot consider themselves productive enough as a result of human resource shortages and that they are concerned about the quality of care provided to patients. Smith's findings (2001) also confirmed the latter results [5].

## Conclusion

This study revealed nurses' views of productivity and human resource factors improving and impeding it. Nurses are fully aware of the importance of the qualitative dimension of productivity and factors contributing to it. The goal of productivity is to promote an adequate and satisfactory level of nursing care acceptable by patients, nurses and physicians [30]. If these findings can contribute to nurses' and managers' improved productivity, not only the patients will benefit but the nurses' quality of work life will also improve. The other feature of productivity improvement is its positive effect on attitude of patients, personnel, managers, nurses' job satisfaction, improved morale and budget control.

Managers who are aware of nurses' viewpoints would be able to create conditions to achieve higher productivity levels. The findings are also beneficial to managers, enabling them to identify and promote productive work practices among clinical staff.

Although a wide range of participants' views has been studied in this research, the small number of informants may limit the generalizability of the results.

As health care organizations have a dynamic nature, further studies of productivity trends and the changing nature of its contributing factors is strongly desirable. Furthermore, it is recommended that comparable studies be carried out in different parts of the country, together with quantitative studies to reinforce the studies' results.

## Competing interests

The author(s) declare that they have no competing interests.

## Authors' contributions

NDN: Initiation and design of the research, collection and analysis of the data and writing the paper.

AAN: Main supervision of the project, and editorial revision of draft papers.

MAH: Supervision of the project, co-analysis of the data and editorial revision of draft papers.

FA: Supervision of the project, co-analysis of the data and editorial revision of draft papers.
